# Bilateral Obstructing Ureteroceles Presenting As Hypertension in a Young Adult Without Urinary Symptoms: A Case Report

**DOI:** 10.7759/cureus.115037

**Published:** 2026-08-23

**Authors:** Johnathan C Kao, Alisa C Chalmers, Esther S Yu

**Affiliations:** 1 Department of Medicine, UCLA Health, Los Angeles, USA

**Keywords:** hypertension, orthotopic ureterocele, secondary hypertension, ureterocele, young hypertension

## Abstract

We present a 35-year-old man with long-standing untreated hypertension who had no urinary symptoms, flank pain, or history of urinary tract infections and was later found to have bilateral ureteroceles with bilateral hydronephrosis and renal cortical thinning. A ureterocele is a congenital cystic dilation of the distal ureter as it inserts into the bladder that can impede urinary drainage. The initial laboratory workup for secondary hypertension was unremarkable, and he had a minimal response to first-line blood pressure management. Imaging was then sought to evaluate for structural causes of secondary hypertension, which ultimately revealed the bilateral ureteroceles. This case represents only the second reported instance of bilateral ureteroceles discovered during evaluation for secondary hypertension in an adult. The absence of classic urological symptoms despite significant obstructive uropathy emphasizes the importance of maintaining a broad differential diagnosis when evaluating hypertension in young adults. Structural urological abnormalities, including congenital anomalies, should be considered, as they may represent potentially reversible causes of secondary hypertension.

## Introduction

Hypertension is a highly prevalent condition encountered in primary care around the world, including in young adults [1]. While prevalence increases with age, studies have shown that approximately one in eight people between the ages of 20 and 40 years have hypertension [2]. Despite multiple available treatments, blood pressure control continues to be a challenge in the primary care setting, with some cases likely due to undiagnosed secondary hypertension. Secondary hypertension accounts for approximately 30% of cases in patients under 40 years of age, with the most frequent causes being primary aldosteronism, renovascular hypertension, and primary kidney disease [2]. Current guidelines recommend that all patients with hypertension under 40 years of age should be screened for secondary causes, particularly when hypertension is severe, resistant to treatment, or occurs in the absence of a family history. This workup includes screening for primary aldosteronism, pheochromocytoma, Cushing disease, renal artery stenosis, obstructive sleep apnea, and thyroid disease [2-4]. While this evaluation focuses on endocrine disorders and renovascular disease, structural urological abnormalities represent an often-overlooked category of potentially reversible causes [4,5]. We present this patient as a case of secondary hypertension caused by bilateral ureteroceles in the bladder.

## Case presentation

A 35-year-old African American man presented to the primary care clinic to establish care and for management of long-standing hypertension. A blood pressure of 144/82 mmHg was noted at age 17, and since then, subsequent in-office readings have consistently shown systolic blood pressures of >130 mmHg. He had never been treated with antihypertensive medications. He also denied any history of pyelonephritis, recurrent urinary tract infections, flank pain, hematuria, or lower urinary tract symptoms. Furthermore, he had no known family history of polycystic kidney disease or other hereditary renal conditions. His body mass index was 25.86 kg/m^2^. Physical examination was unremarkable aside from elevated blood pressure. He exercised at least six days a week with a mix of yoga, aerobic, and strength training exercises. He denied significant caffeine use and had no history of anxiety or depression.

Given his young age, a workup for secondary causes was initiated by his previous physician one year prior. Laboratory evaluation included an electrolyte panel, serum aldosterone and plasma renin activity, fractionated plasma metanephrines, and thyroid-stimulating hormone levels. All results were within normal limits (Table 1). A urinalysis was also obtained and showed no abnormalities. Despite counseling regarding the long-term cardiovascular risks of untreated hypertension, the patient declined pharmacologic therapy.

Upon returning to care, his blood pressure was measured at 138/63 mmHg. After discussion of treatment options, he agreed to initiate therapy and was started on losartan 25 mg daily. At his follow-up appointment four weeks later, his blood pressure remained elevated at 150/55 mmHg despite medication adherence.

**Figure 1:**
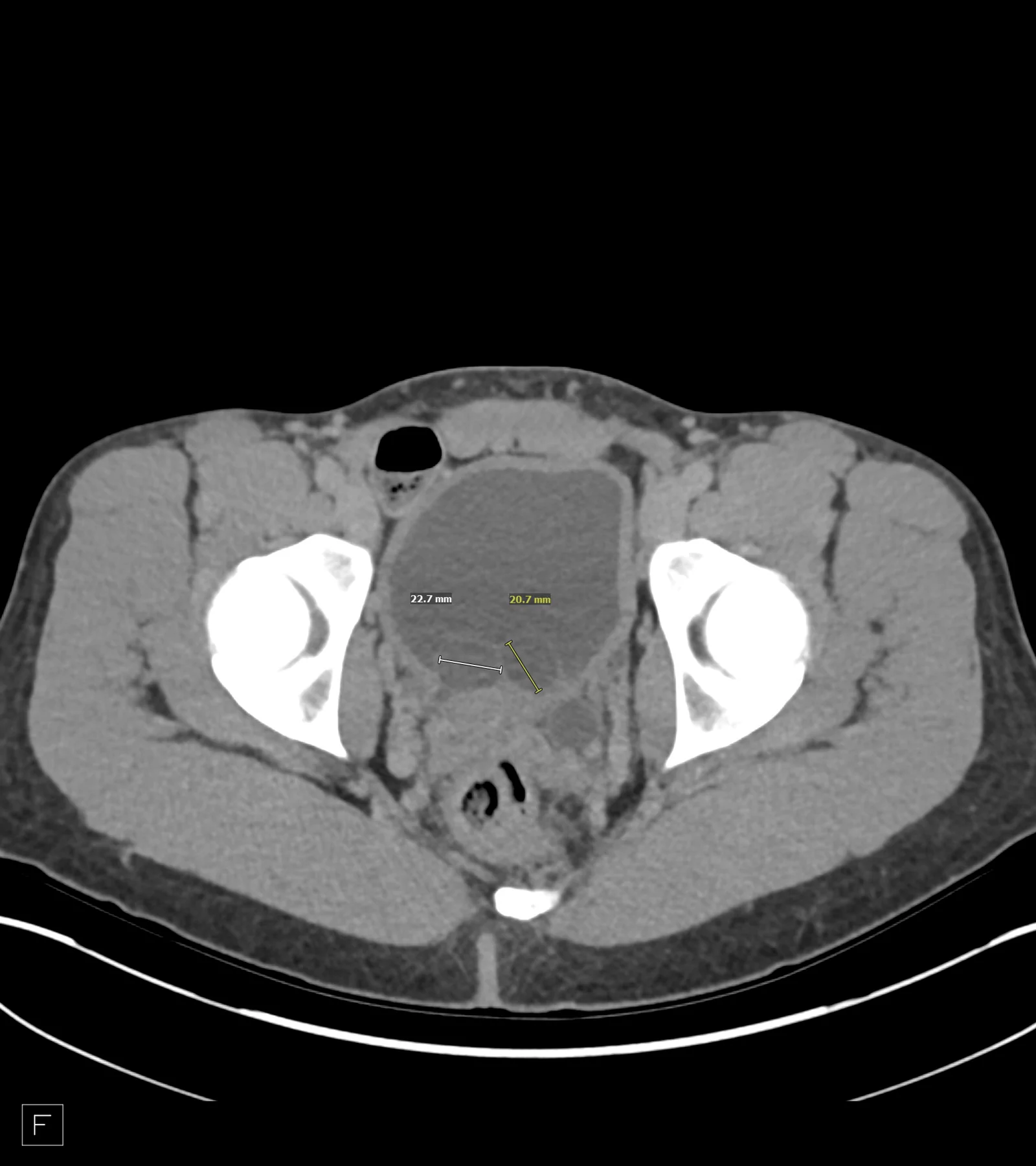
CT urography demonstrating bilateral intravesical ureteroceles at the distal ureters within the bladder.

Given the persistently elevated systolic blood pressure in a young patient despite angiotensin receptor blocker therapy, further evaluation for renovascular hypertension was pursued. Renal artery duplex ultrasonography was ordered and revealed no evidence of renal artery stenosis; however, bilateral hydronephrosis was incidentally noted. Subsequent CT urography was obtained and demonstrated marked bilateral hydroureteronephrosis with associated renal cortical thinning and bilateral 2 cm obstructing intravesical ureteroceles with orthotopic ureteral insertions (Figure 1).

The patient subsequently underwent laser incision of the bilateral ureteroceles with successful drainage of the obstructed collecting systems. However, his blood pressure remained elevated after surgery, and repeated kidney ultrasounds following the procedure showed persistent severe hydronephrosis in the left kidney and mild hydronephrosis on the right with bilateral renal cortical thinning. Laboratory studies obtained four months after treatment were normal except for a mildly elevated serum creatinine (Table 1). His blood pressure was eventually controlled with the use of losartan, amlodipine, and hydrochlorothiazide.

**Table 1:** Laboratory studies obtained during initial evaluation for secondary hypertension and four months after surgical treatment of the bilateral ureteroceles.

	Initial labs	Labs after surgery	Reference range
Sodium (mEq/L)	142	142	135-145
Potassium (mEq/L)	4.3	4.2	3.5-4.5
Chloride (mEq/L)	103	103	101-111
Carbon dioxide (mEq/L)	31	26	21-31
Creatinine (mg/dL)	1.1	1.27	0.6-1.3
Estimated glomerular filtration rate (mL/min/1.73 m^2^)	89	75	>89: Normal; 60-89: Normal to mildly decreased; 45-59: Mildly to moderately decreased; 30-44: Moderately to severely decreased; 15-29: Severely decreased; <15: Kidney failure
Thyroid-stimulating hormone (mcIU/mL)	1.42		0.35-4.0
Aldosterone (ng/dL)	3		Upright 8:00-10:00 am: ≤28; Upright 4:00-6:00 pm: ≤21; Supine 8:00-10:00 am: 3-16
Renin activity (ng/mL/hr)	0.82		0.25-5.82
Plasma metanephrines (nmol/L)	0.31		0-0.49
Plasma normetanephrine (nmol/L)	0.39		0-0.89

## Discussion

Ureteroceles are cystic dilations of the distal ureter that can occur fully within the bladder (intravesical) or extend partially or completely external to the bladder neck (ectopic) [6]. Autopsy data suggest a prevalence as high as 1 in 500, though studies evaluating the incidence of clinically significant ureteroceles in pediatric admissions report rates of 1 in 5,000 to 1 in 12,000, indicating a wide spectrum of clinical disease [7]. The pathophysiology of ureteroceles remains incompletely understood, and no single embryologic theory has adequately explained the variation in ureterocele types and presentations. Most cases are diagnosed antenatally on prenatal ultrasonography, while urinary tract infections in the pediatric population often serve as a sentinel event prompting diagnosis [6]. Adult presentation is rare and typically manifests with recurrent urinary tract infections, incontinence, or lower urinary tract symptoms rather than obstructive uropathy.

The association between obstructive uropathy and hypertension is well established. Ureteral obstruction activates the renin-angiotensin-aldosterone system, increases renal sympathetic nerve activity, and induces oxidative stress, all of which contribute to elevated blood pressure [8,9]. Importantly, relief of bilateral ureteral obstruction has been shown to result in rapid improvement or resolution of hypertension, underscoring the reversible nature of this mechanism when identified and treated promptly [10]. However, with prolonged obstruction, chronic kidney disease can develop, which also leads to hypertension [1]. In these cases, renin and aldosterone levels are often normal, though the exact mechanism of this is unknown, as over-activation of the renin-angiotensin-aldosterone system is still considered to play a role given the benefits of angiotensin-converting enzyme inhibitors in preventing chronic kidney disease progression [11]. Given the duration of obstruction and bilateral renal cortical thinning noted on this patient's imaging, the current hypertension may be more related to chronic kidney disease rather than obstructive uropathy alone, further emphasizing the importance of early identification of secondary hypertension when possible reversible causes can be addressed.

Prior to this case, only one other report has described incidental bilateral ureteroceles in an adult discovered during evaluation for secondary hypertension without urologic symptoms. In that case, a 36-year-old man was found to have bilateral hydronephrosis with marked renal cortical thinning. He was treated with point diathermy, resulting in an 8% improvement in renal function. Subsequent blood pressure control, however, still required the use of oral antihypertensive medications [12]. Our patient similarly presented without classic urological symptoms despite significant bilateral hydroureteronephrosis and renal cortical thinning. Despite treatment of the bilateral ureteroceles, follow-up kidney ultrasounds showed persistent bilateral hydronephrosis and renal cortical thinning, and he also required the use of oral antihypertensive medications to maintain blood pressure control.

This case highlights several important clinical considerations. First, the absence of urinary symptoms and normal routine laboratory findings in our patient emphasizes the importance of maintaining a broad differential diagnosis when evaluating hypertension in young adults. Second, the incidental discovery of bilateral hydronephrosis on renal artery ultrasonography--obtained to evaluate for renovascular disease--underscores the value of imaging in the workup of secondary hypertension, even when the initial indication differs from the ultimate diagnosis. Third, timely endoscopic intervention may allow for relief of obstruction and prevent progression to chronic kidney disease and persistent hypertension.

## Conclusions

In conclusion, bilateral obstructing ureteroceles represent a rare but potentially reversible cause of secondary hypertension in adults. Treatment of hydronephrosis has been shown to reverse hypertension. However, in both this case and the previously reported case of ureterocele-associated hypertension, treatment of the ureterocele did not lead to a return to normotension, likely due to the delayed diagnosis resulting in prolonged untreated hydronephrosis and renal scarring. These cases emphasize the importance of early recognition and intervention in secondary hypertension to preserve renal function and improve blood pressure control, particularly in younger patients and those who do not respond to appropriate pharmacologic therapy.
